# Long-Term Follow-Up of Restorations of Equine Cheek Teeth Infundibula (2006–2017)

**DOI:** 10.3389/fvets.2021.793631

**Published:** 2022-01-14

**Authors:** Christopher J. Pearce, Nicky Brooks

**Affiliations:** Equine Dental Clinic Ltd, Wimborne, United Kingdom

**Keywords:** infundibulum, infundibula, restoration, hypocementosis, caries

## Abstract

**Background:**

Caries of the infundibula of equine cheek teeth can lead to significant dental disease including increased attritional wear, pulpar and apical disease, secondary sinusitis, and dental fracture. Restorations of cavities of equine cheek teeth infundibula have been performed since 1889. Recent advances in dental materials, instrumentation, and techniques have facilitated the use of dental restoration techniques by equine veterinary practitioners. No studies to date have demonstrated the safety or efficacy of restorations of equine cheek teeth infundibula.

**Objectives:**

To assess the long-term results of restorations of equine cheek teeth affected by infundibular caries, to report on the safety of the procedure, and to give guidelines for future restorative therapies.

**Study Design:**

Retrospective analysis of results of clinical and oroscopic examination of horses that underwent infundibular restoration procedures between 2006 and 2017.

**Methods:**

A total of 223 infundibula in 185 maxillary cheek teeth in 92 horses were restored using a variety of dental materials including glass ionomer cement, flowable and compactible resin composites. The time between restoration and re-examination was recorded along with findings of clinical signs in the interim, restorative material loss, and any further pathological changes of the teeth including caries progression, fracture, or apical disease. Follow-up examinations were performed over two study periods 2006–2012 and in 2017.

**Results:**

Over the full study period, 99% of treated horses available for follow-up examinations had no adverse clinical signs or developed any abnormalities of restored teeth observable on oroscopic examination. Of horses re-examined, 83% of restorations were shown to have minimal or no loss of the restoration material, with occlusal surface wear visibly comparable to other adjacent maxillary teeth. Statistical analysis showed success of the procedure was related to the restorative material used, the restoration technique, and the caries grade present at the time of restoration (grade 3 is more successful than grade 2).

**Main Limitations:**

There are no case controls in this study and therefore it is not clear if restoration of equine infundibula is a consistently beneficial procedure, or at which grade of caries progression restorations should be performed for optimum benefit. The procedures were not re-examined at consistent regular times creating some difficulties in standardizing results. Re-examinations of treated horses did not consistently include radiography or computed tomography and therefore some apical changes may have occurred in treated teeth without visual oroscopic or external clinical signs.

**Conclusion:**

Restoration of equine infundibula using materials developed for human dentistry including flowable resin composites is a safe and long-lasting procedure and appears to prevent the development of further pathological changes including apical infection and dental fracture.

## Introduction

**Figure 1:**
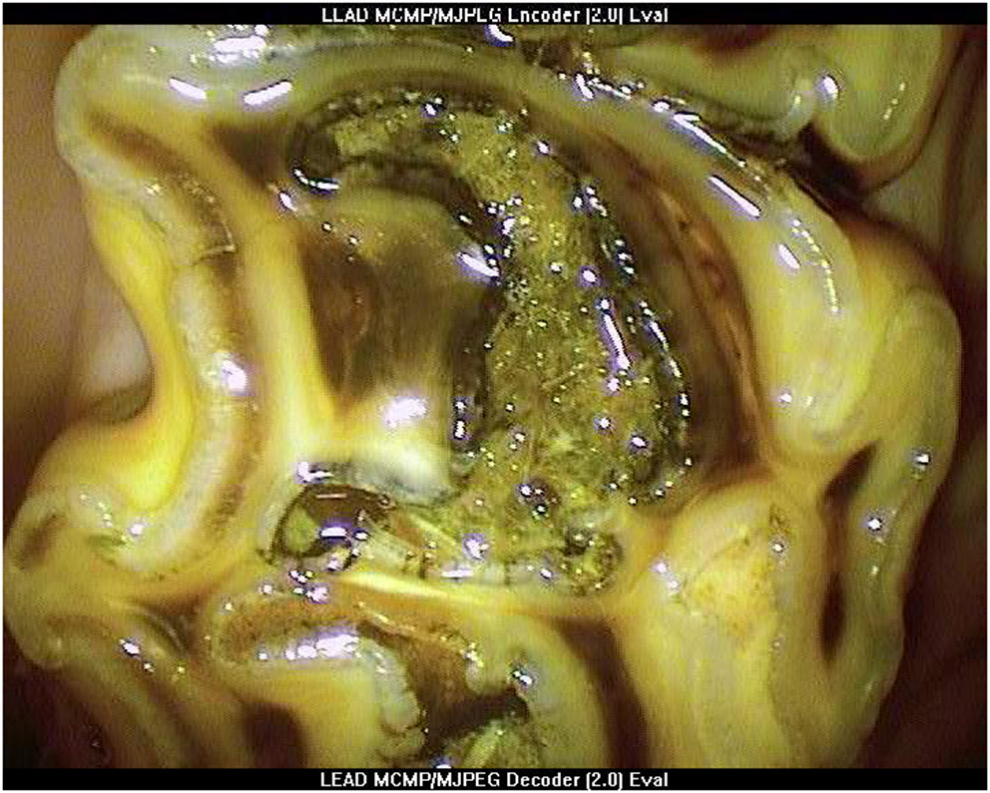
Oroscopic image of Grade 3 mesial infundibular caries of a Triadan 109 tooth at presentation.

The equine dental infundibulum (plural: infundibula) is anatomically an invagination of the dental enamel from the occlusal surface in an apical direction that is present in incisors (*n* = 1) and maxillary cheek teeth (*n* = 2) (1). The vascular supply to the developing infundibulum is from its occlusal aspect, through the central infundibular artery, and via small side vessels mesially and distally (2). The infundibula of erupted cheek teeth are technically inert, although the lateral vascular supply usually survives for a short time (months to years) post eruption (3, 4). Abnormalities of the infundibula of maxillary cheek teeth include developmental and acquired disorders (5), the most common being variable degrees of infundibular hypocementosis (IH) (2, 4, 6) and infundibular caries (IC) (5, 7–12). Infundibular hypocementosis most commonly affects the apical aspect of the infundibulum although rarely there may be more complete or even total absence of cementum (4, 13). Prevalence of infundibular abnormalities has been shown to be up to 90% in anatomical (14), clinical (15), and computed tomographic (CT) (4, 5) studies. In one study 90% of teeth showed significant infundibular changes on combined occlusal surface and CT examination (16). Another study showed a similar prevalence with only 11.7% of infundibula of clinically normal horses completely filled with normal appearing cementum on CT examination (4). Other earlier studies also described similar ranges of prevalence of infundibular disorders (17, 18). Dental caries has been defined as a progressive acidic demineralization of the inorganic matrix of dental tissues secondary to bacterial fermentation of carbohydrate substrate and subsequent organic matrix loss (19, 20). A likely etiology is that exposure of IH lesions occlusally through normal physiological dental eruption and attrition results in impaction of food ingesta into the exposed infundibular defects, resulting in secondary caries (Figure 1) (11, 12, 21–23). In Sweden infundibular caries of the Triadan 106/206 positions were found to be strongly associated with the presence of a novel bacteria (*Streptococcus devreisei*) isolated from caries lesions (20). Subsequent molecular bacteriology has shown hundreds of bacterial species including Streptococci to be present in dental disease, but their individual roles are difficult to elucidate (24).

Infundibular caries appears to be variably progressive, depending on the extent and the site of the IH lesion and thus the age of occlusal exposure of the infundibular defect. Potential sequelae include increased occlusal attrition (22), pulpitis and apical infection (13), and dental fracture (10, 13, 17). Increased attrition is considered due to loss of infundibular cementum and most importantly enamel. Microbial invasion from the carious infundibulum to the dentine-pulp complex is the likely cause of apical infection (3, 13, 25). One study showed caries from the infundibulum caused apical sepsis in 16% of maxillary cheek tooth apical infections (13); however, this has subsequently been suggested to be an underestimate (3). Advanced lesions of both mesial and distal infundibula, especially those that coalesce, are likely to result in significant structural weakness of the tooth (12, 26). Infundibular caries related dental fracture is assumed to be due to IC providing a central plane of structural weakness in the tooth thus predisposing it to pathological fracture when exposed to the normal forces of mastication (27). Studies have repeatedly demonstrated that the maxillary Triadan 09 position is most commonly affected by infundibular hypocementosis, caries, and secondary associated sequelae (4, 10, 13, 15, 17, 18, 28, 29). One study found caries affecting the full length of the infundibulum in 8.2% of infundibula studied, most commonly in the 12–20-year age group, and with the average depth of 35 mm in carious infundibula (4).

The principles of dental restoration are to “protect the pulp, arrest decay, restore the tooth to function, and prevent further disease” (30). Restorations of human teeth dates to Neolithic times (31) with the practice becoming widespread through the late 19th century (32). Documentation of equine dental infundibular restorations is sparse but dates to 1889 (33, 34).

## Methods

Teeth that had undergone infundibular restorations by the author between 2006 and 2012 were re-examined clinically and oroscopically during this period, and then again during 2017. All oroscopic examinations were performed under standing sedation with video recordings of treated teeth.

### Case Selection for Infundibular Restoration

Teeth were selected for restoration during examinations at routine or referral dental appointments. Grading of infundibular caries was performed using a classification system modified from an original system by Honma (35). The grading system used is outlined below:

Grade 1—Caries of infundibular cementum only.
Grade 2—Caries of infundibular cementum and enamel.
Grade 3—Caries of infundibular cementum, enamel, and dentine.
Grade 4—Caries of mesial and distal infundibula with coalescence.
Grade 5—Infundibular associated dental fracture, apical disease, or tooth loss.

At the initial examination, teeth considered potentially suitable for restoration were assessed oroscopically using the following selection criteria and using the above grading system:

1. Grade 3 infundibular caries with a minimum infundibular defect depth of 10 mm (Figure 1) or,
2. Grade 2 infundibular caries if contralateral to a tooth with grade 5 infundibular caries, minimum infundibular defect depth 10 mm and,
3. Absence of clinical evidence of pulpar disease of the selected tooth, or of ipsilateral sinusitis or a history of ipsilateral nasal discharge or malodor.

Initially, all cases had routine radiography of their maxillary cheek teeth. However, after ~50 cases, radiography was only utilised for horses that presented with or had a history of ipsilateral nasal discharge or had teeth that had clinical evidence of pulp involvement oroscopically (secondary dentine defects, fractures, gingival draining tracts, or focal gingival recession). Teeth with these changes were not selected for restoration.

### Restoration Procedure

**Figure 2:**
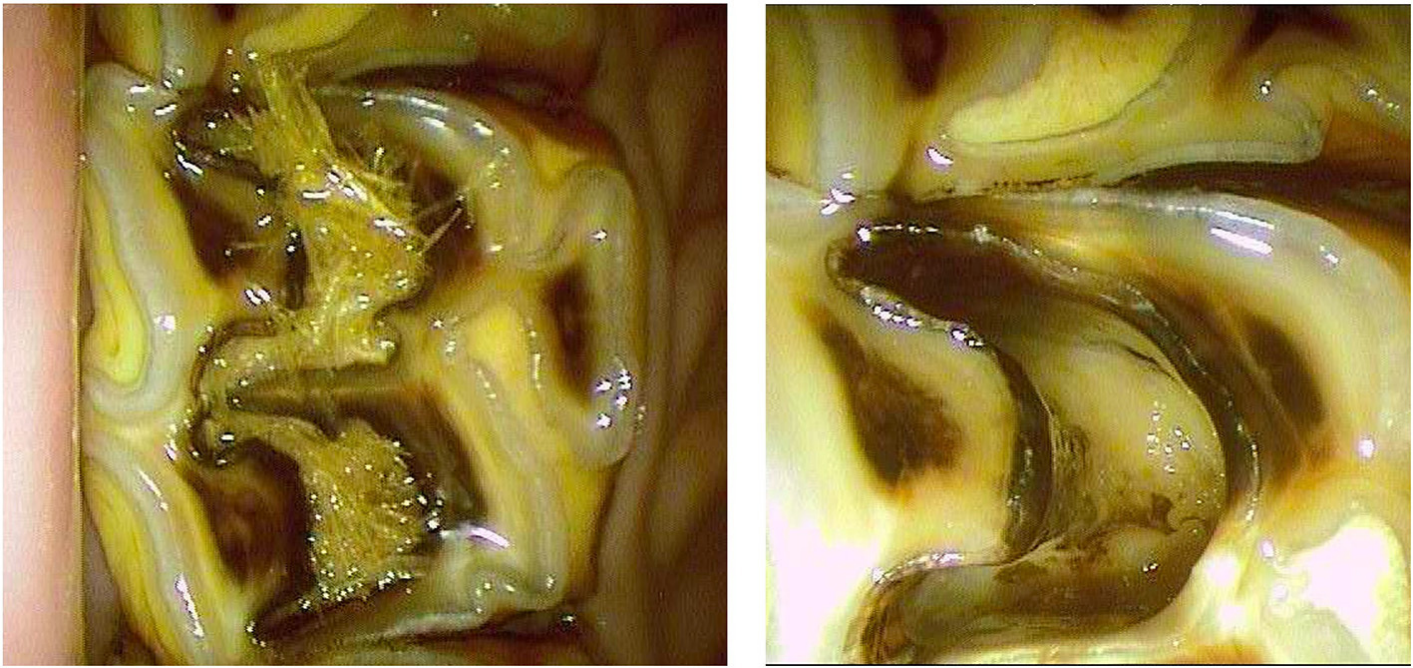
Oroscopic image showing preparation of mesial and distal infundibula of a Triadan 109 tooth prior to restoration. *Left*, before and *Right* after successful cavity preparation.

**Figure 3:**
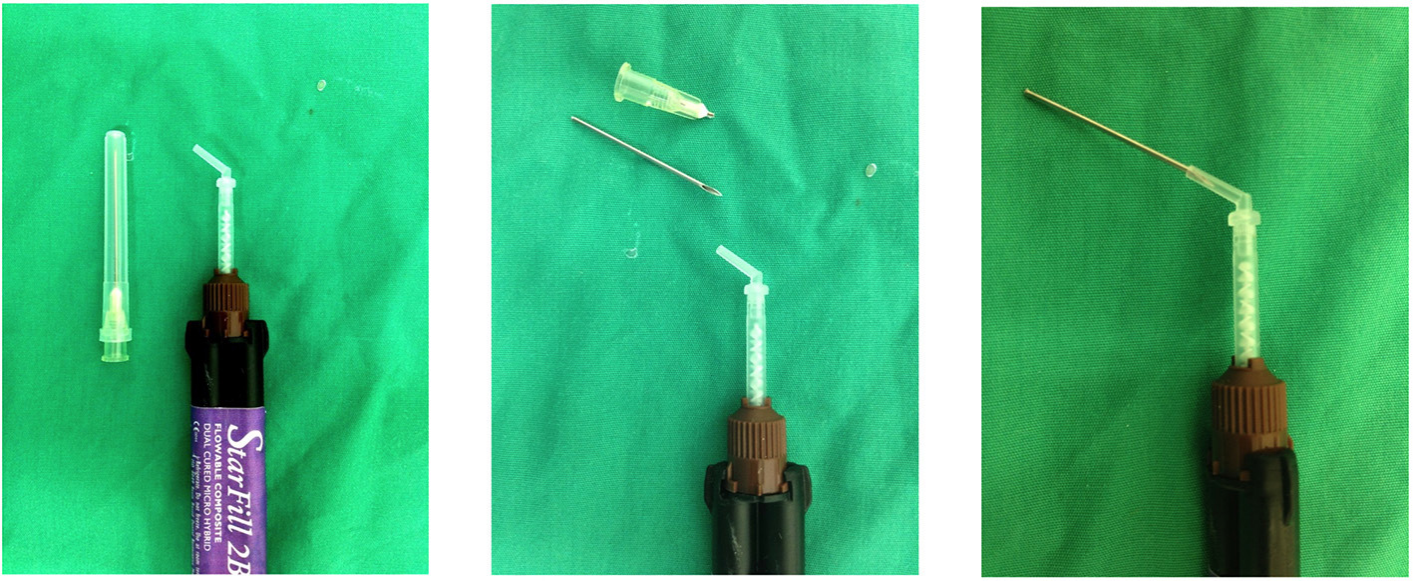
Method used to fabricate mixing tip extensions using a 19G 3.8 cm hypodermic needle.

All procedures were performed under standing sedation and oroscopic guidance. A pneumatic high speed, water irrigated turbine handpiece, with handpiece extensions for equine use, various burrs, and a 90-degree high pressure air and water wand were used for all cavity preparations (Equine Perio-unit, Veterinary Dental Products, Wisconsin). During the 6 years (2006–2012) of the first follow-up period, small alterations to the technique were made as the author became more experienced with the equipment and procedure, along with input from human dentists and veterinary dental specialists. A summary of the technique is outlined below:

1. Impacted food was removed from the occlusal aspect of infundibular cavities (defects) using a sharp occlusal probe or K-flex file (Sybron Endo, CA) held in hemostats.
2. Deeper impacted food and carious cementum or dentine was removed as far apically as possible using burrs and a high-speed dental handpiece, under continuous or intermittent oroscopic guidance. Burr types and sizes ranged from surgical length (29 mm) no. 8 round carbide or diamond burs, cylindrical diamond burs (29 mm) to surgical taper fissure burs (31 mm). In some cases, a slow-speed handpiece with a 15 mm right-angle (RA) to friction grip (FG) mandrel adaptor (Premium Plus UK Ltd, Dorset, England) was used, using one of the FG burs above, thus increasing the total burr length up to 45 mm.
3. Any remaining deeply impacted food material and debris was manually removed from the infundibular apex using K-flex files and Hedstrom files (60 mm, Dr Shipps, USA). A carbamide peroxide-EDTA file lubricant gel (Canal Plus, Septodont, UK) was used to coat the file or was injected apically into the infundibulum to aid the debridement procedure.
4. The cavity was flushed with high pressure air and water to remove the loosened products of the above debridement (in cases where patency of the infundibular apex was not suspected).
5. The infundibular cavity was inspected oroscopically, and Steps 2–4 were repeated until the cavity walls and the infundibular apex were visually clear of all debris and carious material as far as was possible. The cavity was recorded as fully debrided when the apex and walls were clearly visible with no remaining impacted food or discolored cementum remaining (Figure 2), or as not fully debrided, if some such inaccessible material remained prior to restoration.
6. The cavity was then flushed with 4.5% NaOCl, 2% chlorhexidine, or water (details recorded), rinsed with water, and air dried.
7. A single-etch bonding product was applied thinly to the cavity walls (Adper Prompt L-Pop, 3M), thinned with air (to distribute it more evenly and avoid clumping of liquid), and then light cured for 20 s.
8. The cavity restoration was performed (under oroscopic control) using a variety of materials and techniques:
  a. Glass ionomer cement applied manually and compacted using a metal compactor and burnisher (Fuji IX, GC, Tokyo, Japan).
  b. Compactible composite applied as above (XOP, R&S Dental, Paris, France).
  c. Dual-cured flowable resin composite (Starfill, Danville, USA) placed either in an incrementally layered filling technique (see below), or bulk filled without any light cure, using a 19G 38 mm needle tips reversed and inserted into the mixing tip (Figure 3).
  d. Combinations of the above (compactible composite used as a base liner, followed by occlusal flowable resin composite).

### Flowable Composite Application Techniques

Flowable resin composite restorative material (dual-cured) was placed either using a modified oblique incremental layered technique based on a human technique (36) or as a bulk fill. For the incremental layered technique, material was placed from the apical aspect occlusally, in 3–4 layers, filling the narrowest parts of the cavity first, usually the infundibular buccal infoldings. This was to utilise the increased surface tension of placing a viscous liquid material in the narrow margins of the cavity, to avoid gravitational slumping (flow ventrally). A further 2–3 layers were then applied in the central region of the defect, working occlusally. A short light cure was occasionally required to prevent occlusal gravitational flow of the material. For bulk fill restorations, the material was simply extruded in an apical to occlusal direction, starting centrally and filling the entire cavity continuously, while moving the needle tip in short, rapid occlusal to apical motions to help drive the material into the narrow margins of the cavity. No light cure was applied for bulk fill restorations.

All restorations were performed at a single session. Referral cases were only included in the study if the first author was able to personally re-examine the cases. Cases were re-examined oroscopically at varying time intervals following treatment over a 6-year period (2006–2012) and those available for further follow-up were subsequently re-examined during 2017. For the first dataset (2006–2012) results were collated and analysed statistically. For the second data set, the occlusal oroscopic appearance of the restoration was recorded but no statistical analysis of results was performed due to the large number of cases lost to follow-up.

### Statistical Analysis

Data from the first period of follow-up was statistically analysed using Pearson chi-square to test for independence between sets of two variables; the *P*-value was set at 0.05 or less when the null hypothesis that the variables were independent of each other could be rejected, demonstrating that the variables were related. Success was defined as the tooth still present and with more than 90% of restorative material remaining occlusally at oroscopic examination, no fracture of the tooth, no immediate evidence of complications related to the procedure, and no clinical signs of dental disease (sinusitis, facial swelling, dysmastication) since treatment.

Statistical analyses examined the success of the procedure in relation to Triadan number, age of horse, grade of infundibular caries prior to restoration, restorative material used, mesial vs. distal infundibulum, restoration technique (incremental layered restoration vs. bulk fill), and whether the infundibular apex was completely debrided prior to restoration.

## Results

**Figure 4:**
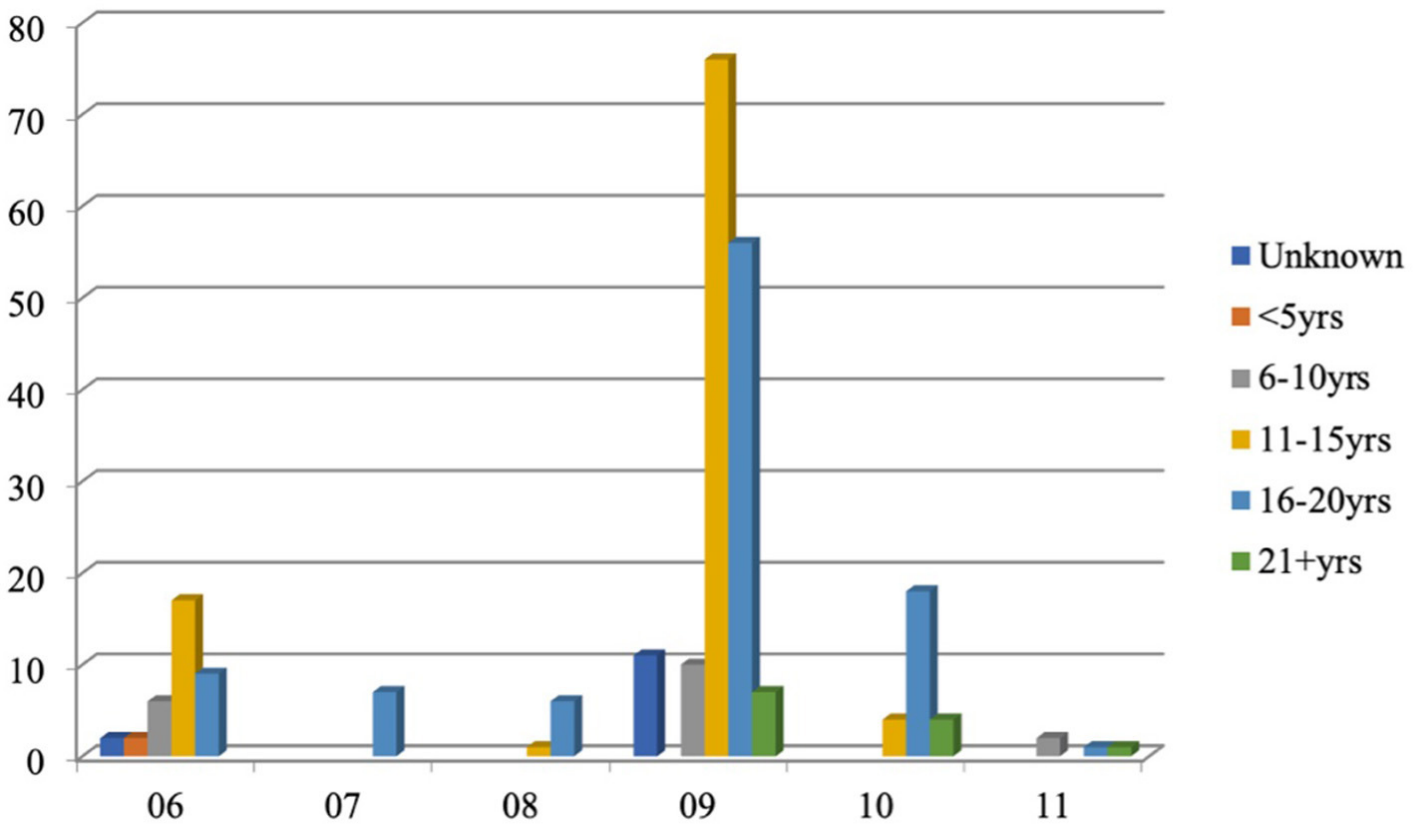
Number of teeth by Triadan position and ages of horses (years) with infundibular caries lesions selected for restoration (left and right sides combined).

A total of 223 infundibula in 185 teeth from 92 horses met the selection criteria and were restored. The mean age of these horses was 14.7 years (range 6–25 years). The number of teeth selected for restoration was similar for the left and right sides. The frequencies of combined left and right Triadan positions and horse age are shown in Figure 4. The Triadan 09 position was the most commonly treated position representing 66.7% of teeth treated; the 06s (15.3) and 10s (10.4%) were the next most commonly treated positions, followed by a very low percentage of 08s (3.2), 07s (3.2), and 11s (1.4%).

Overall, 63% of infundibula selected for restoration were mesial, 5% the distal infundibulum only, 25% mesial and distal infundibula (in the same tooth), and 7% with coalescence of mesial and distal infundibula (grade 4). The grade of infundibular caries selected for treatment was most commonly grade 3, in the maxillary 09 and 06 positions, with the most common grades vs. ages displayed in Table 1.

**Table 1:** Most common grades of infundibular caries (IC) and Triadan positions of teeth restored and age groups of treated horses.

**Age group (years)**	**Number of restorations**	**Percentage of all treated cases**	**Most treated triadan position**	**Most common grade of IC treated**
0–5	2	0.89	06	2
6–10	20	9.38	06, 09	3
11–15	90	40.18	09	3
16–20	91	40.63	09	3
20+	9	4.02	09	3
Age unknown	11	4.91	09	2, 3

The most common material used for restoration was a flowable, dual-cured resin composite (Starfill, Danville, USA) placed directly using either an incremental layered technique (80% of procedures) or a direct bulk fill (10%). Table 2 shows the frequency of materials used.

**Table 2:** Frequencies of materials used for infundibular restorations.

**Restorative material**	**Number of procedures**	**Percentage of procedures**
Starfill resin composite	150	67%
XOP compactible composite	9	4%
XOP (base) + Starfill (occlusal)	41	18%
Fuji IX (base) + Starfill (occlusal)	9	4%
Others	14	7%

Horses were re-examined in two separate periods of follow-up. The initial follow-up period was between 2006 and 2012, with the mean follow-up time during this period being 28 months (range 2–72 months). A further follow-up study was performed in 2017 (mean follow-up time 78 months, range 60–120 months).

**Figure 5:**
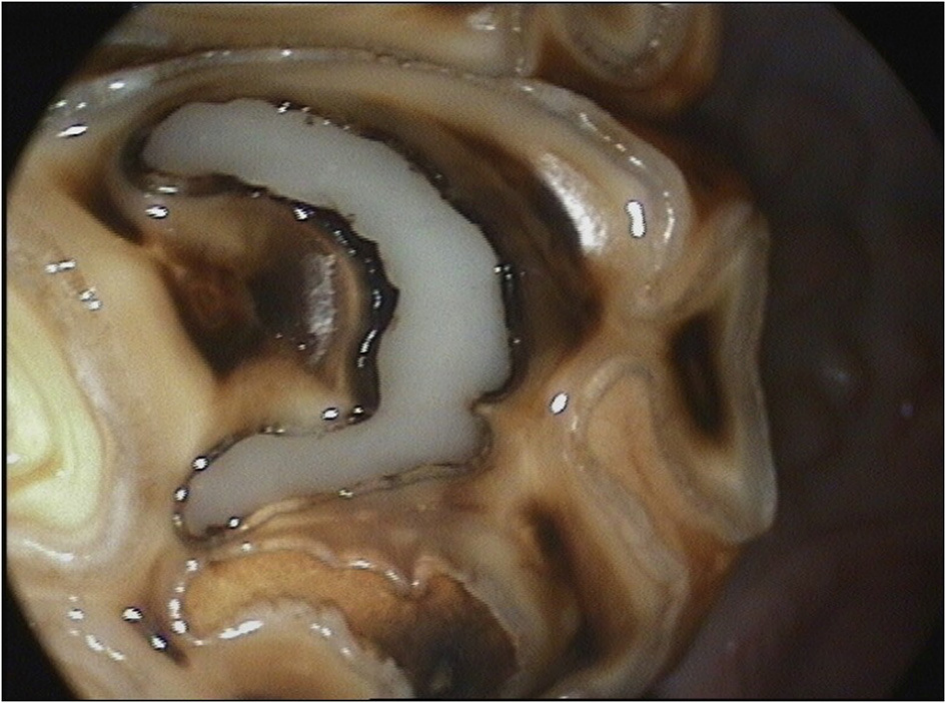
Oroscopic image of the mesial infundibulum of a Triadan 109 tooth, 2 years post-restoration.

**Figure 6:**
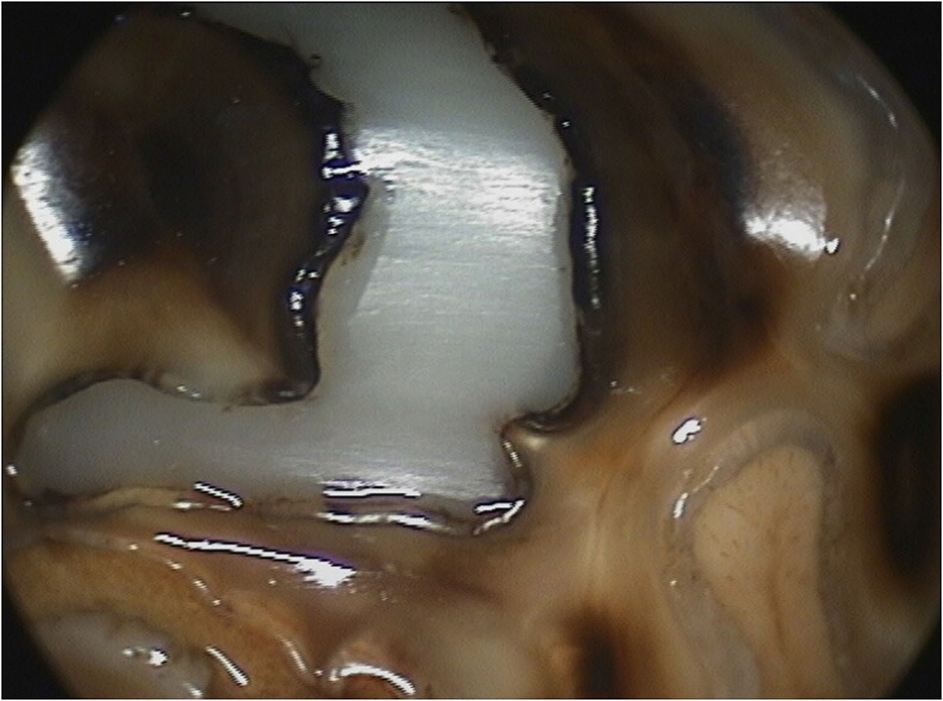
Close up oroscopic images of a Triadan 109 tooth 2 years post-restoration (same tooth as Figure 5). Attritional wear lines can be seen through the restorative material.

**Figure 7:**
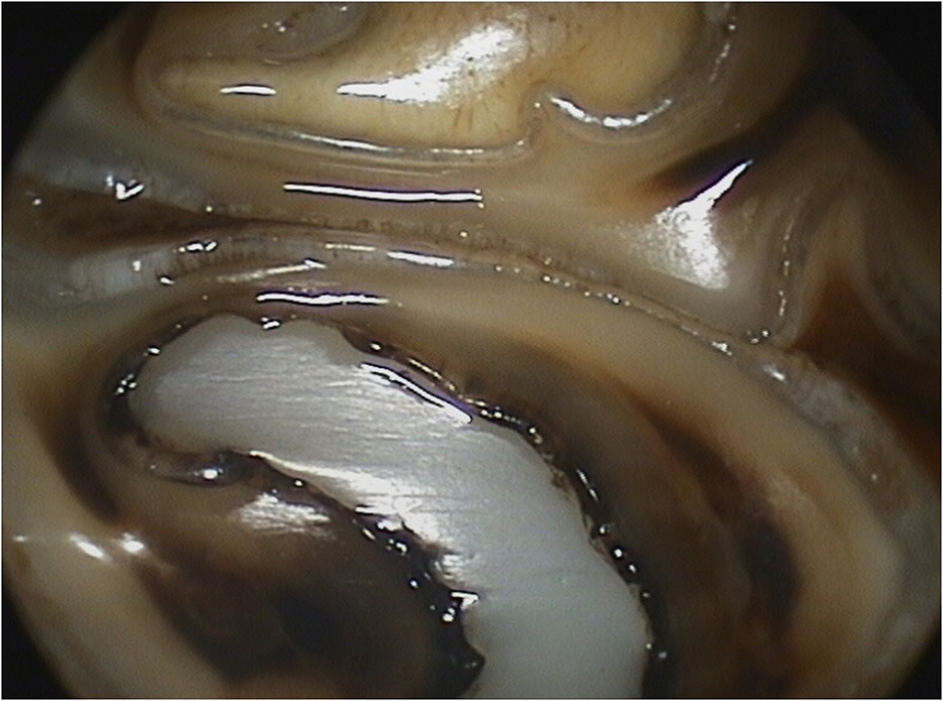
Close up oroscopic images of a Triadan 109 tooth 2 years post-restoration (same tooth as Figure 5). Attritional wear lines can be seen through the restorative material.

**Figure 8:**
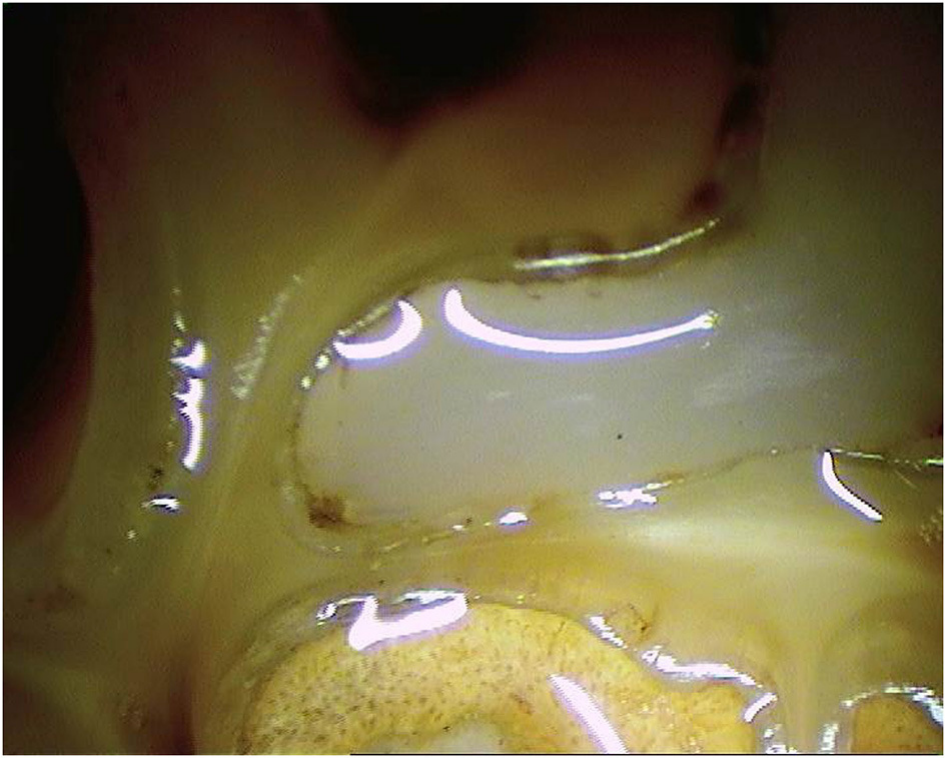
Oroscopic image of the distal buccal infolding of the mesial infundibulum of a Triadan 109 tooth 15 months post-restoration.

**Figure 9:**
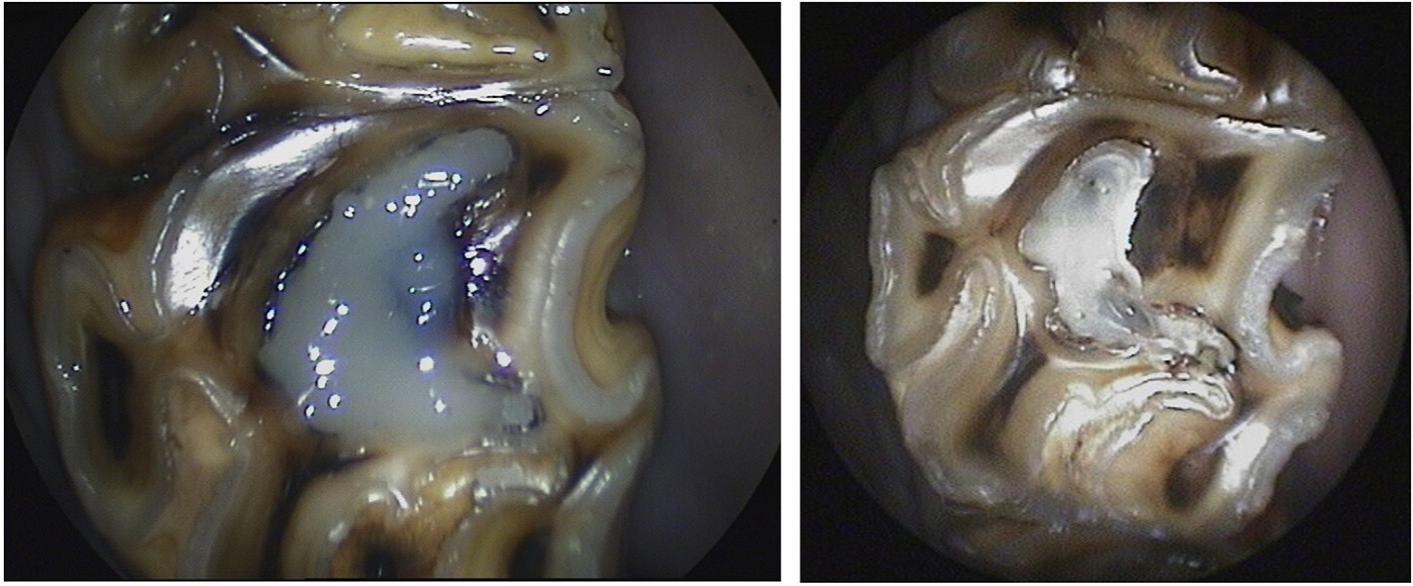
Oroscopic images of a Triadan 209 immediately after (left) and 2 years 4 months after (right) infundibular restoration.

**Figure 10:**
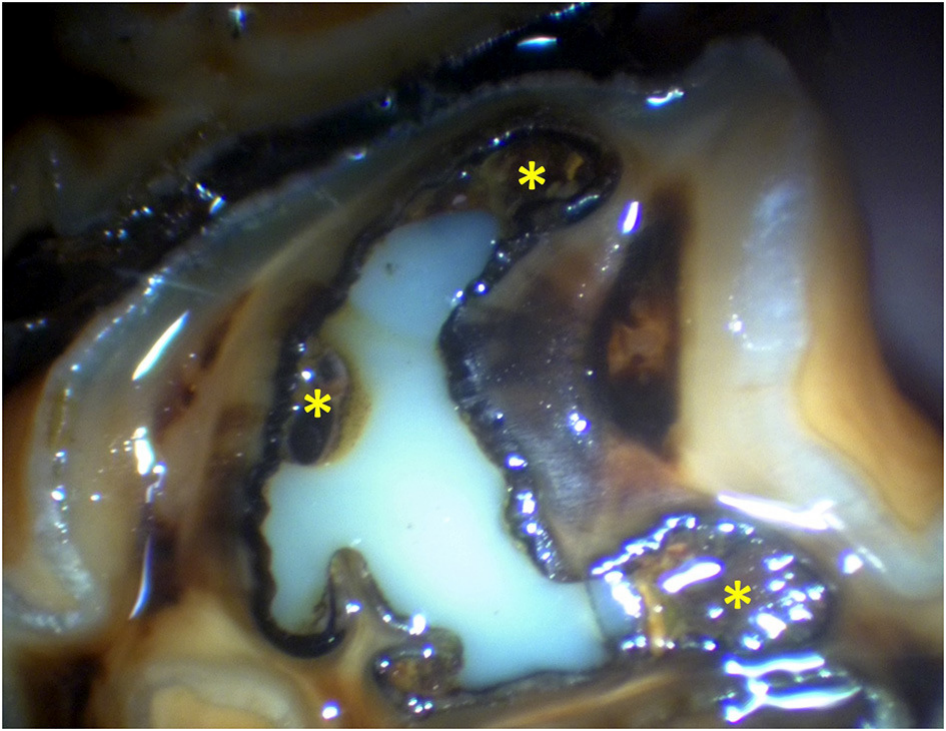
Oroscopic image of the mesial infundibulum of a Triadan 209 4 years 3 months post-restoration of the mesial infundibulum, showing some defects in the restoration (*).

Over the initial study period, 90/92 horses (97%) developed no apparent post-restoration complications in the treated teeth. Two horses developed apical infection post-restoration (one tooth each) and these teeth were subsequently extracted. Restorations were documented to be successful (complete restorations present or <10% of restorative material absent occlusally, with no deterioration of infundibular caries grade) in 193/225 restorations (86%), in 77 horses (83%), with normal occlusal surface wear of the treated teeth, including differential wear of enamel and the restorative material present at re-examination (Figures 5–9). Twenty restorations (10.8% of procedures) from 10 horses had restorations with partial or complete loss, or absence of restorative material (categorized as failures) at re-examination, and in all cases the restoration was replaced as the teeth were still considered vital (Figure 10). Partial crown fractures involving one infundibulum were identified in 6 teeth (3%) prior to restoration with no further fracture later recorded after restoration. Iatrogenic pulp exposure during cavity preparation was identified in one horse that did not receive any specific pulp therapy and without any recorded later complications. Twelve horses (13%) were initially referred for extraction of teeth with infundibular caries related fractures, with other teeth in these horses selected for restorations (25 teeth in total). None of the 25 restored teeth with infundibular restorations had progressed to fracture at the time of follow-up (mean follow-up time 39 months) and with 3/25 teeth (12%) having partial loss of restorative. Four horses (4.3%) with teeth selected for restorations had other teeth identified as having concurrent Grade 1 or 2 lesions which were not restored but which subsequently fractured, with no progression of caries grade or dental fracture occurring in the restored teeth.

From this first study period, statistical analysis was performed on 160 restored teeth for which full data was available (SPSS statistical analysis software, IBM). Results of Pearson chi-squared analysis showed that success was either related to or independent of several factors. Compactible chemical cured composite (“XOP”), compactible glass ionomer cement (“Fuji IX”), and combinations of products had a lower success rate compared to flowable dual cured composite (Starfill) (*P* < 0.0005, 6 d.f). Infundibular caries grade was found to be significant, with grade 2 lesions having a higher failure rate than grade 3 (*P* < 0.005, 4 d.f.). The Triadan position of the restored tooth, the age of the horse, and the site of infundibulum restored (mesial vs. distal) were not related to success (*P* > 0.25). The degree of apical debridement (complete vs. incomplete) was also not found to be related to success (*P* = 0.1, 1d.f).

**Figure 11:**
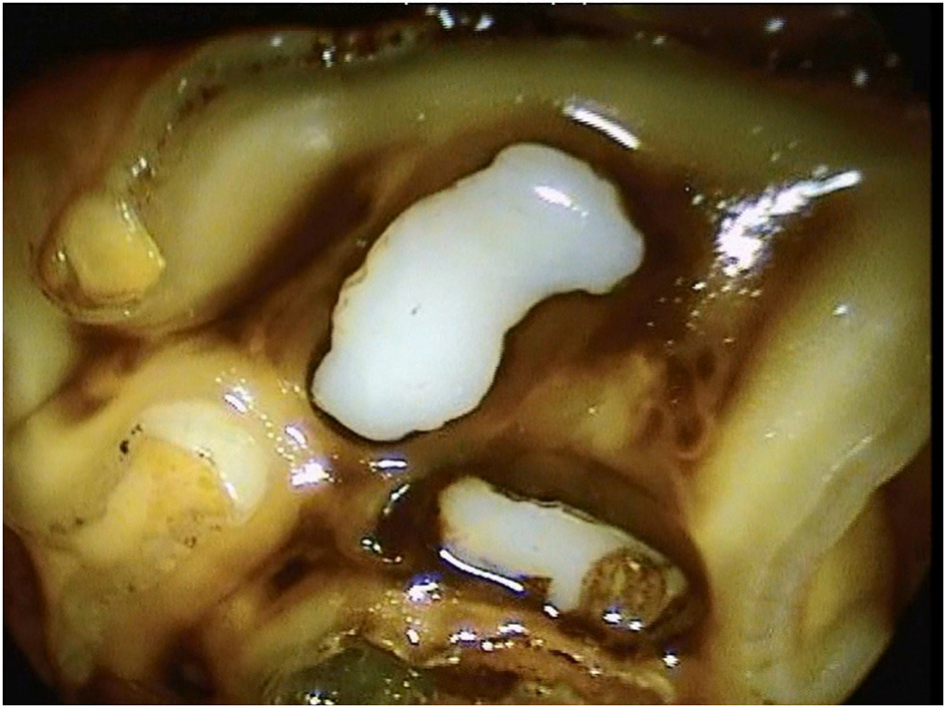
Oroscopic image of a Triadan 209 tooth 10 years post-restoration of the mesial infundibulum.

**Figure 12:**
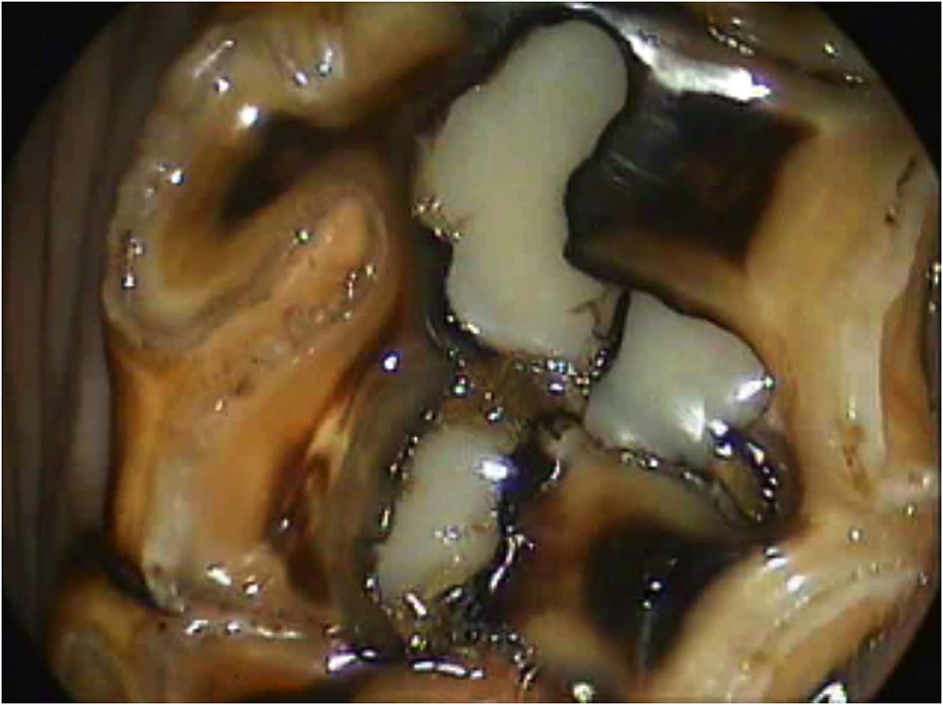
Oroscopic image of a Triadan 209 tooth 9 years post-restoration of the mesial infundibulum.

Out of 92 horses treated initially, 36 teeth in 22 horses were available for a second follow up after a further 5 years (range 60–120 months from initial treatment, mean 78 months). The age range of the horses at this follow-up was 13–27 years, mean age 24 years 4 months. All the 36 treated teeth examined at this stage were present (100%) but one tooth that had been restored 10 years previously now had a midline sagittal fracture. Of the 35 complete teeth, 15 had loss of their infundibula and senile excavation (“cupped” occlusal surfaces) with no filling material (or infundibula) visible. Filling material was still visible in 19 teeth (54% of all teeth, 95% of teeth with infundibula) with 11 teeth (58% of teeth with infundibula) still having complete restorations present on their occlusal surface (Figure 11). Small (1–2 mm) defects containing food material were present at the edges of restorations in seven teeth (37%) (Figure 12). One restoration had no filling material visible, with food material present within the restored infundibulum and an occlusal exposure of pulp horn three. Teeth other than those restored were absent from three horses having fractured due to midline infundibular caries-related sagittal fractures according to the clinical histories.

## Discussion

Infundibular restoration was shown in this study to be a safe technique, with minimal complications recorded post-treatment over a prolonged period. A limitation of the study is that the data does not report the likely outcome had these teeth not been restored, but it appears likely that in the restored teeth the caries was arrested, as almost all the teeth remained structurally sound. Additionally, only 2/160 (0.13%) teeth that were re-examined developed pulp or apical disease as assessed from oroscopic and clinical examinations. Considering the time scale for the follow up examinations (up to 11 years following treatment for the second follow up) it is likely that had there been pulp disease, gross clinical signs, secondary dentine pulp defects, or gingival discharging tracts would have been identified in at least some cases. In two teeth from horses from the second data set that had a single infundibulum restored, secondary dentine defects were observed in these restored teeth, but the affected pulp canals were not adjacent to the restored infundibula, but adjacent to the other carious infundibula that were not restored (did not initially meet the selection criteria). This may suggest that restoration adequately protects the pulp; however, the results are not conclusive due to the low numbers of secondary dentine defects identified in unrestored teeth.

The selection criteria used most frequently selected the mesial infundibulum of Triadan 09 teeth from horses with a mean age of 14.5 years (range 6–25 years). This is consistent with other studies of the prevalence of larger infundibular hypocementosis defects (2, 4, 5, 16) in relation to the expected age of exposure of apical hypocementosis lesions through eruption and attrition, and documented studies of occlusal infundibular caries (7, 15, 24). The cases selected for restoration also share the same age and Triadan position as found in studies of maxillary cheek teeth with fractures and/or apical disease (13, 37, 38). It is not statistically evident from this study that these variables are related, or that restoration of infundibula of these teeth definitively protects them from future infundibular disease. However, studies have shown that infundibular hypoplasia and secondary caries is responsible for apical disease in a significant number of teeth (3). It seems likely as proposed previously, that apical infundibular hypocementosis lesions, described as a developmental malformation of infundibula (3), become more prone to cause caries once these lesions are exposed occlusally (3, 13, 17). If restoration is a safe procedure in the long term, as this study shows, then prevention of food impaction into these defects, thus preventing further caries should protect the endodontic system post-restoration. The results showed that out of all the teeth restored and re-examined later (even up to 11 years later), only 1/185 restored teeth subsequently developed a mid-sagittal infundibular related fracture. There are no controls in this study so it cannot be proven that restorations of carious infundibula prevent fractures, but considering the age range and Triadan number, and advanced caries grades of the restored teeth, these teeth being the most associated with fracture and often complicated extractions (38) it is plausible to consider that restoration provides structural stability to the tooth long term.

The effectiveness of different materials used to restore teeth was shown to vary, with flowable resin composite placed using an incremental layering technique being the most successful. This may be in part due to the operator performing this technique most frequently and therefore developing better material handling skills. It may also be due to the material itself, with consistent observation of this material oroscopically flowing into the infundibular recesses, resulting in a more complete filling and bonding of the restoration material to the infundibular walls. Compactible materials used in human dentistry frequently rely on undercuts to retain them which were not used in these cases, and it is possible that poor compaction technique resulted in less than adequate bonding to the cavity walls in this study.

Documentation of long-term cheek tooth wear with infundibular composite restorations in this study shows that the resin composite used wears down at similar rates to the surrounding dentine, with enamel wearing more slowly than the composite, resulting in enamel ridges protruding slightly from the surface (Figures 5–9). This may be due to the resin composite chosen being a posterior restorative or core build-up material and not generally suitable for human dental crown replacement that requires higher wear resistance. Meta-analysis of studies into longevity of posterior resin composite restorations in humans has shown a mean failure rate of 1.6–4.1% (39), lower than reported in this study. However, direct comparisons to human restorations are difficult due to the huge differences in the cavity sizes in horses compared to humans, infundibular vs. pulp cavity restorations, and the material bonded to (dentine in humans vs. enamel, cementum or occasionally dentine in horses). Also, the human studies generally had different criteria for success to this study, with failures including the later development of dental fracture, progressive caries, or endodontic disease. Using these criteria, our results are easily comparable considering so few of the teeth restored in this study progressed to fracture or endodontic disease. This meta-analysis of human composite restorations (39) also showed that there was a marked increase in failure rate for each extra surface of the restoration exposed orally. Equine infundibular restorations are usually deep intra-coronal restorations, with most of the surface area of the filling bonded to the surrounding tooth, and only a small surface area of the restoration exposed occlusally, therefore having a higher retention rate.

The success of restorations vs. infundibular caries grade at the time of restoration was shown to be higher with grade 3 caries compared to grade 2. This was contradictory to the expected outcome that larger cavities (grade 3) would have greater restoration material loss. The results may be due to the material that the restorative is bonded to, the age of the horse, and therefore the depths of the cavities, or other factors. The single etch product used almost exclusively for these cases is particularly suited to enamel and dentine bonding, but not for cemental bonding, more likely to be employed in cases of grade 2 caries where more infundibulum cementum is present. The open structure of cementum compared to enamel or dentine may collapse when a strong acid etch is used (as for the single etch product used) resulting in bond failure. This is reported in human dentistry, with both single etch adhesive systems and total etch systems being reported to have significantly lower bond strengths to cementum compared to dentine (40). There was no statistical analysis of other factors relating to this and therefore there may be unrecognized factors involved such as the age of the horse and the size of restored infundibula. In some cases, restorations classified as failures were initially considered to be caused by loss of restorative material. However, on re-examination of the original oroscopic images prior to restoration, it is likely instead that some of these were areas of cementum that were not debrided, particularly at the margins of the buccal and the palatal infoldings, which subsequently developed caries when exposed occlusally (Figure 10). In a future development of the technique, the author has made more effort to remove these areas of cementum.

The outcome of infundibular restoration was not statistically linked to the degree of debridement of the apex immediately prior to restoration. This was not as expected, following the assumption that a dental cavity must always be completely clean and free of foreign material for a successful restoration. In preparing these cavities, a 32 mm high speed water irrigated surgical fissure bur was always used to carefully debride the walls of the cavities under oroscopic guidance, followed by manual instrumentation of the more apical aspects, always using a disinfectant lubricant gel. Considerable effort was always made to achieve a fully debrided apex devoid of all impacted food and carious cementum. In some instances, however, it either became too time consuming, or the apical discoloration noted oroscopically was assumed to be cementum that was firmly adhered to the apical enamel and drilling it away with a longer bur may have risked penetration of the apex and exposing pulp, as occurred in one case. In these instances, the cavity was flushed with disinfectant (4.5% NaOCl) before restoration. Reasons for the results showing no difference in outcome could have been the chemical sterilization in the apical region, or the creation of a bacteria-tight seal by adequately preparing the cavity walls to a depth of around 30 mm, using a strong bond effective for enamel, preventing any carbohydrate substrate reaching the apical region. Any viable bacteria present in organic matter trapped apically would be entombed and likely to remain dormant, unable to multiply or cause dental tissue erosion through caries without a supply of carbohydrate from the occlusal aspect. This is presumably why the very high prevalence of apical infundibular hypocementosis with little or no occlusal communication is generally considered little more than an incidental finding, and why infundibular lesions with apical enlargement and significant occlusal communication (16) can lead to progressive and serious caries (3, 25). In subsequent developments of the technique of infundibular restoration, the author has used a two-stage endodontic style technique utilizing calcium hydroxide paste for a temporary period to disinfect and clean the apical regions in such cases more effectively (T. Lundstrom, personal communication) (41).

In summary, intra-coronal direct restoration of carious infundibula using a flowable dual-cured resin composite, bonded using the single etch process, was shown to be a safe long-term procedure with minimal loss of restorative material, and minimal clinical disease of treated teeth. The case selection criteria used in this study selected teeth that have been shown in other studies to be high risk for fracture and apical disease, and restorations of these infundibula may prevent these possible pathological sequelae. Further controlled studies, and studies using ancillary imaging modalities such as radiography and computed tomography of restored teeth will help to further qualify the safety and efficacy of this procedure.

## Data Availability Statement

The raw data supporting the conclusions of this article will be made available by the authors, without undue reservation.

## Ethics Statement

Ethical review and approval was not required for the animal study because the horses in this study underwent clinical procedures that make up part of standard clinical practice and no procedures were performed that were not considered necessary or ethical. Written informed consent for participation was not obtained from the owners because the clients / owners of these horses signed consent forms for the procedures which were performed as part of clinical practice.

## Author Contributions

CP was the primary author who selected the clinical cases, performed the clinical procedures, and wrote the manuscript. NB performed the follow-up of the second case series in 2018 recording the cases oroscopically and collated the data. All authors contributed to the article and approved the submitted version.

## Conflict of Interest

The authors declare that the research was conducted in the absence of any commercial or financial relationships that could be construed as a potential conflict of interest.

## Publisher's Note

All claims expressed in this article are solely those of the authors and do not necessarily represent those of their affiliated organizations, or those of the publisher, the editors and the reviewers. Any product that may be evaluated in this article, or claim that may be made by its manufacturer, is not guaranteed or endorsed by the publisher.
